# Nutritional status and post-operative complications in patients undergoing surgery for advanced pharyngeal or laryngeal cancer

**DOI:** 10.1007/s00405-023-08139-x

**Published:** 2023-08-03

**Authors:** Teresa Brown, Anna Edwards, Alice Pashley, Belinda Lehn, Sarju Vasani, Robert Hodge, Judith Bauer

**Affiliations:** 1 School of Human Movement and Nutrition Sciences, University of Queensland, Brisbane, QLD Australia; 2 Department of Dietetics and Food Services, Royal Brisbane and Women’s Hospital, Brisbane, QLD Australia; 3 Department of Nutrition and Dietetics, Toowoomba Hospital, Darling Downs Health, Toowoomba, QLD Australia; 4 Department of Speech Pathology, Royal Brisbane and Women’s Hospital, Brisbane, QLD Australia; 5 Department of Ear Nose Throat, Royal Brisbane and Women’s Hospital, Brisbane, QLD Australia; 6 Department of Nutrition, Dietetics and Food, Monash University, Melbourne, VIC Australia

**Keywords:** Head and neck cancer, Nutrition, Post-operative complications, Malnutrition, Pressure injury

## Abstract

**Purpose:**

Malnutrition is an important prognostic indicator of post-operative outcomes in patients undergoing surgery for head and neck cancer, however, limited studies utilize validated nutrition assessment tools to accurately assess risk. The aim of this study was to determine the relationship between nutritional status on post-operative complications and length of stay for patients undergoing either a laryngectomy, pharyngectomy or pharyngolaryngectomy for head and neck cancer.

**Methods:**

Patients with head and neck cancer undergoing a laryngectomy, pharyngectomy or pharyngolaryngectomy at a tertiary hospital in Australia were eligible for this retrospective cohort study (*n* = 40). Nutritional status was assessed by the dietitian on admission using the validated Subjective Global Assessment tool. Clinical outcomes were collected via retrospective chart review and included length of stay and post-operative complications.

**Results:**

Pre-operative malnutrition incidence was 40%. Malnourished patients had higher incidences of any type of complication (57% vs 44%, *p* = 0.013) and pressure injury (86% vs 14%, *p* = 0.011) compared to well-nourished patients. Well-nourished patients had a clinically important shorter median length of stay compared to malnourished patients (17.5 vs 20 days).

**Conclusion:**

Early identification and management of malnutrition is essential to minimize risk of post-operative complications and reduce length of stay and should be considered a key component of prehabilitation programs.

## Introduction

Head and Neck Cancer (HNC) refers to malignancies of the oral cavity, pharynx, larynx, nasal cavity, paranasal sinuses, and the major salivary glands [1]. Over the last 20 years, there have been advances in the management of HNC, such as microscopic and endoscopic surgery, robotic surgery for oropharyngeal resections, intensity modulated radiotherapy techniques, and the development of targeted agents and immunotherapy [2, 3]. For patients with tumors involving the larynx ± pharynx; laryngectomy or pharyngolaryngectomy are common surgical procedures, however, are often associated with significant morbidity [4]. Key indications for a laryngectomy are advanced stage primary disease, salvage procedures for recurrence after chemoradiotherapy and/or significant laryngeal dysfunction associated with aspiration after chemoradiotherapy. Minimally invasive transoral robotic surgery for laryngectomy is not yet established as standard treatment in routine clinical practice and thus total laryngectomy via open surgery remains a relevant procedure in modern HNC management [5].

Malnutrition is also common in patients with HNC, with multifactorial causes such as lifestyle factors, tumor obstruction impeding oral intake and side effects from multimodality treatments [6, 7]. Malnutrition has long been identified as an important prognostic factor, associated with poorer quality of life and reduced survival in patients undergoing radiotherapy [8, 9], as well as being associated with post-operative complications including the development of pharyngocutaneous fistula, infection, and delayed wound healing [10–12].

The Global Leadership Initiative on Malnutrition (GLIM) recommendations state that malnutrition should be diagnosed by a combination of at least one phenotypic (weight loss, low body mass index, or reduced muscle mass) and one etiologic criterion (reduced food intake/assimilation or inflammation) [13]. Evidence-based practice guidelines for the nutritional management of adult patients with HNC recommend the use of a validated nutrition assessment tool for assessing and diagnosing malnutrition, such as the Subjective Global Assessment (SGA) and related Patient-Generated Subjective Global Assessment (PG-SGA) [14, 15]. These tools meet the key diagnostic features for malnutrition presence as suggested by the GLIM criterion and have been used as predictors of prognosis in oncological surgical procedures [16–18]. To our knowledge, no studies have investigated the effects of malnutrition diagnosed using a validated nutrition assessment tool on post-surgical complications for patients with HNC undergoing a total laryngectomy or pharyngolaryngectomy.

The aim of this study was to firstly assess the incidence of malnutrition as determined by a validated assessment tool (SGA) in a surgical HNC population undergoing either a laryngectomy, pharyngectomy or pharyngolaryngectomy; and secondly determine the relationship between nutritional status on post-operative complications and length of stay (LOS).

## Methods

This retrospective study was conducted between 2012 and 2014 at a large tertiary hospital in Australia. All patients with diagnosed HNC of the pharynx or larynx undergoing either a laryngectomy, pharyngectomy or pharyngolaryngectomy were included.

A prospective database was established by the Department of Speech Pathology to assess speech and swallowing outcomes in this patient population and monitor post-operative complications. Baseline and outcome data were recorded prospectively from medical records. Retrospective chart review was performed to confirm missing data and accuracy of data collection. Ethics was obtained through relevant hospital Human Research Ethics Committees (Reference LNR/2019/QRBW/52094).

Patient demographics included age, sex, and diagnostic information (Tumor, Node, Metastasis (TNM) stage [19], tumor location according to the International Classification of Diseases, 10th revision [ICD-10]), and treatment details (i.e. flap reconstruction type and any previous chemotherapy or radiotherapy). Comorbidities were recorded for calculation of Charlson’s Comorbidity Index (CCI) [20]. Baseline nutritional status was collected for each patient on admission as part of standard dietetic care using SGA [21]. Weight, height and BMI were recorded based on reference categories taking into account age at diagnosis [22]. Diet texture modification on admission was categorized as full, soft, minced, puree, or nil by mouth (NBM) and fluid prescription was recorded as thin fluids, thick fluids or NBM.

Postoperative complications during the admission and post-discharge period for up to 30 days were recorded; including wound site infection, development of fistula, delayed wound healing/wound dehiscence, failed swallow due to anastomotic leaks, pressure injuries and any others. Length of stay (LOS) was recorded in days, with an extended LOS defined as longer than the upper interquartile range of the median.

Data were presented as mean (standard deviation, SD) or median (interquartile range, IQR) for continuous variables, and categorical variables presented as count (%) throughout. To examine the characteristics of the sample population, descriptive statistics were used. Patients were divided in two groups based on nutritional status, either as well nourished (SGA A) or malnourished (SGA B or C); and where possible other categorical variables were collapsed into categories for statistical analysis. Pearson’s *χ*^2^ or Fischer’s exact test was used to determine relationships between categorical variables. Two-sample *t* tests were used to determine relationships between categorical variables and continuous variables or the Wilcoxon Rank Sum Test for non-normally distributed data. Statistical significance was set with a *p* value < 0.05. Data were analyzed using R Commander (R version 3.6.1 (2019-07-05)).

## Results

**Fig. 1:**
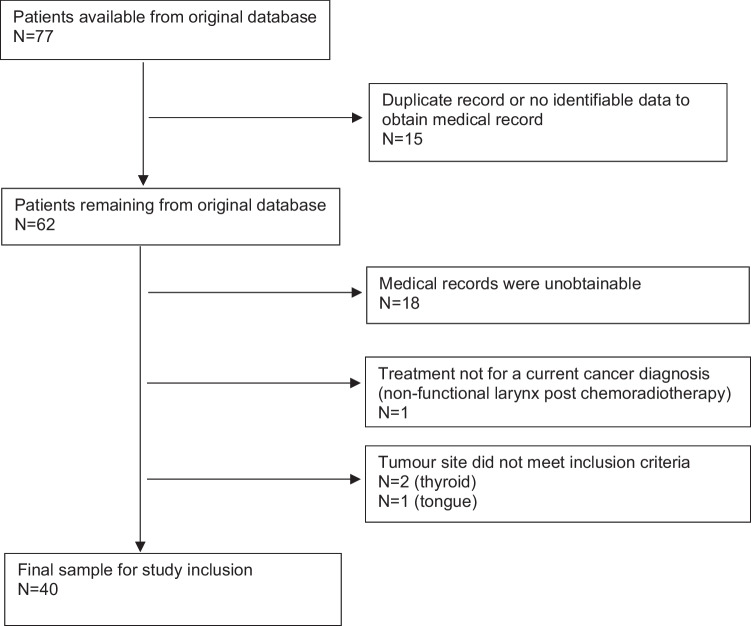
CONSORT flow diagram for patient selection

In total, 40 patients were eligible for inclusion in the analysis (Fig. 1), with patient characteristics shown in Table 1. There were no significant differences between the malnourished and well-nourished patients. Mean age was 66.7 (9.7) years and were predominantly male (*n* = 36, 90%). The most common diagnosis was advanced stage squamous cell carcinoma of the larynx. Salvage surgery was performed in 18 patients who had previous radiotherapy/chemotherapy treatment (45%).

**Table 1:** Patient demographics and clinical characteristics

Variable	Characteristic	Total, *N* = 40	Nourished, *N* = 24 (60%)	Malnourished, *N* = 16 (40%)	*P* value
Sex^a^	Male	36 (90)	22 (61)	14 (39)	1
	Female	4 (10)	2 (50)	2 (50)	
Age^b^	Mean years (SD)	66.7 (9.7)	66.6 (9.4)	66.9 (10.4)	0.913
Cancer site^a^	Hypopharynx	8 (20)	2 (25)	6 (75)	0.066
	Supraglottis	7 (17.5)	4 (57)	3 (43)	
	Larynx	25 (62.5)	18 (72)	7 (28)	
T stage^a^	T1	3 (7.5)	3 (100)	0 (0)	0.109
	T2	10 (25)	7 (70)	3 (30)	
	T3	11 (27.5)	8 (73)	3 (27)	
	T4	16 (40)	6 (37.5)	10 (62.5)	
N stage^a^	N0	24 (60)	16 (67)	8 (33)	0.554
	N1	4 (10)	2 (50)	2 (50)	
	N2	12 (30)	6 (50)	6 (50)	
Histology^a^	Squamous Cell Carcinoma	39 (97.5)	23 (59)	16 (41)	1
	Chondrosarcoma	1 (2.5)	1 (100)	0 (0)	
Surgery^a^	Laryngectomy	26 (65)	17 (65)	9 (35)	0.292
	Pharyngo- laryngectomy	12 (30)	5 (42)	7 (58)	
	Pharyngectomy	2 (5)	2 (100)	0 (0)	
Flap^a^	No flap	26 (65)	17 (65)	9 (35)	0.746
	Anterolateral thigh	3 (7.5)	1 (33)	2 (67)	
	Radial artery forearm	2 (5)	1 (50)	1 (50)	
	Jejunal	9 (22.5)	5 (56)	4 (44)	
Prior radiotherapy^c^	Yes	18 (45)	12 (67)	6 (33)	0.436
Prior chemotherapy^a^	Yes	5 (12.5)	2 (40)	3 (60)	0.373

^a^Fishers exact test

^b^Two-sample *t* test

^c^Chi Squared Test

Patient baseline nutrition assessment data are shown Table 2. In total, 16 (40%) patients were malnourished prior to surgery, with 11 (27.5%) mild to moderately malnourished (SGA = B) and 5 (12.5%) severely malnourished (SGA = C). According to BMI category, 14 (36%) patients were underweight. Prior to surgery, 47.5% (*n* = 19) required some degree of diet/fluid texture modification and seven patients (17.5%) were placed NBM. Both peri-operative diet and fluid modifications were associated with malnutrition (*p* = 0.007 and *p* = 0.017 respectively).

**Table 2:** Patient characteristics for baseline nutritional assessment

Variable	Characteristic	Total, *N* = 40	Nourished, *N* = 24 (60%)	Malnourished, *N* = 16 (40%)	*P* value
BMI category^a,b^	Underweight	14 (36)	4 (29)	10 (71)	0.007*
	Healthy weight	10 (26)	6 (60)	4 (40)	
	Overweight/obese	15 (38)	13 (87)	2 (13)	
Diet texture^b^	Full diet	14 (35)	11 (79)	3 (21)	0.007*
	Soft diet	9 (22.5)	8 (89)	1 (11)	
	Minced diet	4 (10)	2 (50)	2 (50)	
	Puree diet	6 (15)	2 (33)	4 (67)	
	Nil by mouth	7 (17.5)	1 (14)	6 (86)	
Fluid modification^b^	Thin fluids	30 (75)	21 (70)	9 (30)	0.017*
	Thickened fluids	3 (7.5)	2 (67)	1 (33)	
	Nil by mouth	7 (17.5)	1 (14)	6 (86)	

^*^Statistically significant value

^a^Missing data *n* = 1

^b^Fishers Exact Test

**Fig. 2:**
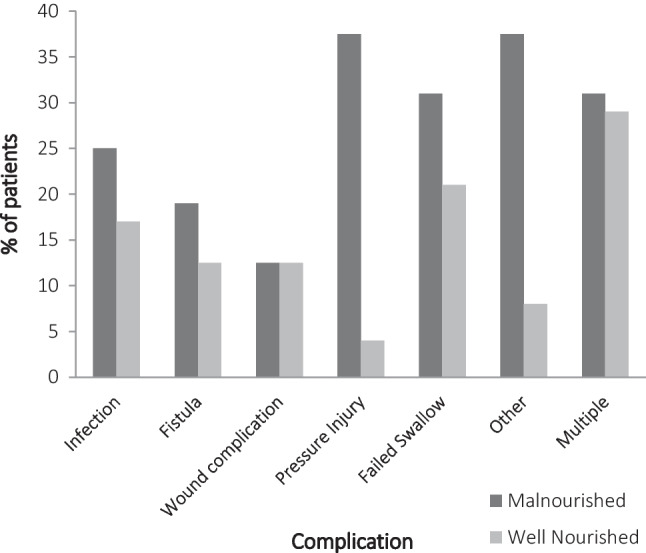
Post-operative complication incidence according to baseline nutritional status

The incidence of post-operative complications is summarized in Fig. 2 and Table 3; with 57.5% (*n* = 23/40) of patients affected by at least one complication, with a higher incidence in the malnourished group versus the well-nourished group (*p* = 0.013). There was also a significant difference found for pressure injuries (*p* = 0.011) and other complications (haematoma (*n* = 3), cardiorespiratory (*n* = 3), delirium (*n* = 1), and ileus post jejunum harvest (*n* = 1); *p* = 0.042). Four patients (10%) required an unplanned ICU admission for the management of their complications. Wound-healing incidence was similar in each group at 12.5% each. There was a clinically important higher infection rate in the malnourished group (25%) versus the well-nourished group (17%), but this did not reach statistical significance (*p* = 0.691).

**Table 3:** Post-operative complication incidence (N, %) according to nutritional status

Category	Total (*N* = 40)	Nourished (*N* = 24)	Malnourished (*N* = 16)	*P* value
Any complication^a^				0.013*
Yes	23 (57.5)	10 (42)	13 (81)	
No	17 (42.5)	14 (58)	3 (19)	
Infection^b^				0.691
Yes	8 (20)	4 (17)	4 (25)	
No	32 (80)	20 (83)	12 (75)	
Fistula^b^				0.668
Yes	6 (15)	3 (12.5)	3 (19)	
No	34 (85)	21 (87.5)	13 (81)	
Wound-healing delays^b^				1.00
Yes	5 (12.5)	3 (12.5)	2 (12.5)	
No	35 (87.5)	21 (87.5)	14 (87.5)	
Pressure injury^b^				0.011*
Yes	7 (17.5)	1 (4)	6 (37.5)	
No	33 (82.5)	23 (96)	10 (62.5)	
Fail swallow/anastomotic leak^b^				0.482
Yes	10 (25)	5 (21)	5 (31)	
No	30 (75)	19 (79)	11 (69)	
Other complications^b,c^				0.042*
Yes	8 (20)	2 (8)	6 (37.5)	
No	32 (80)	22 (92)	10 (62.5)	
Multiple complications^b^				1
Yes	12 (30)	7 (29)	5 (31)	
No	28 (70)	17 (71)	11 (69)	
Extended length of stay^b^				0.482
Yes	10 (25)	5 (21)	5 (31)	
No	30 (75)	19 (79)	11 (69)	

^*^Statistically significant value

^a^Chi squared

^b^Fishers exact test

^c^Other complications: haematoma (*n* = 3); cardiorespiratory (*n* = 3); delirium (*n* = 1); ileus post jejunum harvest (*n* = 1)

Approximately, 42% (*n* = 10/24) of well-nourished patients and 81% (*n* = 13/16) of malnourished patients experienced any form of complication post-operatively. Malnourished patients were more likely to develop any complication than those who were well-nourished prior to surgery (*χ*^2^ = 6.155, *p* = 0.013). Simple logistic regression showed that patients who were malnourished were over six times more likely than well-nourished patients to develop any complication (Odds Ratio [OR] 6.1, 95% CI 1.4 to 27.0; *p* = 0.018).

Approximately 4% of well-nourished patients (*n* = 1/24) and 38% of malnourished patients (*n* = 6/16) had a pressure injury or had progression of pressure injury staging. Malnourished patients were more likely to have pressure injuries than those well-nourished prior to surgery (*p* = 0.011). Simple logistic regression showed that patients who were malnourished were approximately fourteen times more likely than well-nourished patients to have a pressure injury (Odds Ratio [OR] 13.8, 95% CI 1.5 to 130.0; *p* = 0.022).

Malnourished patients were more likely to develop other complications than those well-nourished prior to surgery (*p* = 0.042). Simple logistic regression showed that patients who were malnourished were approximately seven times more likely than well-nourished patients to develop other complications (odds ratio [OR] 6.6, 95% CI 1.1 to 38.6; *p* = 0.036). No other complication type was significantly associated with nutritional status.

There was a clinically important longer median length of stay for patients who were malnourished at 20 days (IQR 15.5–25.3) versus 17.5 days (IQR 14.8–21.0) (*p* = 0.274). In total, 21% of patients who were well-nourished (*n* = 5/24) and 31% patients who were malnourished (*n* = 5/16) experienced an extended LOS for their surgical admission (*p* = 0.482). For all outcomes, the sample size was too small to perform multivariable analysis as the models did not converge.

## Discussion

This study aimed to be one of the first to determine the incidence of malnutrition (using a validated nutrition assessment tool) and any associations with clinical outcomes post-laryngectomy, pharyngectomy and pharyngolaryngectomy in a HNC population. There was an overall higher incidence of most complications in patients who were malnourished and a significant relationship between malnutrition and any complication, other complications, and pressure injuries. The difference in median LOS of 2.5 days between patients who were malnourished versus well-nourished was also deemed clinically important.

The incidence of malnutrition on admission was 40%, falling within ranges described previously for HNC [23]. The overall complication rate was 58%, with 81% of patients who were malnourished and 42% of patients who were well-nourished suffering from a post-operative complication. Gourin et al. found 22% of malnourished patients experienced a complication versus 10% of well-nourished patients (in a similar HNC surgical population including laryngectomy and pharyngolaryngectomy procedures) [25]. These rates are lower than our findings, which may be explained by variations in definitions of complications, differences in study inclusion criteria and the method of measuring nutritional status. Most studies have used single parameter measures, such as biochemical markers or anthropometry alone [11, 12, 24, 25], as an indicator of overall nutritional status, rather than a validated tool using the GLIM criteria.

Malnutrition is a well-known predictor of pressure injury development seen in multiple populations [26]. This study found an overall incidence rate for pressure injury to be 17.5%, with a higher incidence in malnourished patients (38%) versus 4% in well-nourished patients (*p* = 0.011). A similar pressure injury incidence of 17.3% was also reported in a mixed cancer population who were admitted for hospice care [27]. One study specific to surgical HNC patients reported an incidence of 14.8% for the development of intraoperative pressure injuries typically located over bony prominences (buttocks or sacrum, *n* = 7; shoulder or back, *n* = 4; ankle or heel, *n* = 3) [28]. They determined that age and longer operative duration were significant factors in the development of pressure injuries in these patients. It is also possible that pressure injuries reported in our study may have been present on admission. A recent study of patients admitted to acute care, found that 7.4% had a pressure injury on admission, and poor nutrition accounted for 41% of cases [29]. Given our study population had a high rate of malnutrition on admission, it can be hypothesized that they likely had existing pressure injuries too.

The extra 2.5 days of hospitalization experienced by patients who were malnourished pre-operatively in this study was deemed clinically important. Not only has LOS been used as a surrogate marker for a patient’s quality of life [30], having extended LOS presents additional costs to the health care system. In addition, if the patient required an admission to ICU to manage their complication, as was seen in some of the cases in this study, then this would also increase the cost of healthcare for that patient [31]. Leandro-Merhi et al. [32] investigated the effects of nutritional status on LOS in an adult and elderly general surgical population. They found patients who were malnourished, compared to patients who were well nourished (diagnosed using the SGA), had a longer duration of hospital stay, similar to the findings of the current study.

Enhanced recovery after surgery (ERAS) protocols, are well established in colorectal surgery guidelines to improve post-operative outcomes [33]. ERAS guidelines have also been developed for the perioperative management of HNC and include aspects of preadmission education and optimisation of preoperative nutritional status [34]. These pre-operative interventions alongside other multimodal and multidisciplinary approaches to optimize patient’s physical, nutritional and mental status prior to surgery has been termed prehabilitation and can also include exercise programs and dysphagia rehabilitation [35, 36]. A systematic review of nutrition interventions undertaken in the HNC prehabilitation setting identified only two studies which examined the use of an enriched formula versus standard nutrition support in malnourished patients and found no additional benefit of the enriched formula in mitigating weight loss [37]. The authors concluded, however, that due to the malnutrition risks on diagnosis and the negative impact of poor nutritional status on clinical outcomes, further robust nutritional prehabilitation research is required to inform clinical practice. Likewise, the findings from our current study reinforces the importance of the identification of malnutrition in patients with HNC prior to surgery so that individualized nutrition intervention can be commenced early to improve post-operative outcomes. This is also in line with the European Society for Parenteral and Enteral Nutrition (ESPEN) guidelines for clinical nutrition in surgery [38] and provides further justification for the role of prehabilitation.

A major strength of this study is that it is the first study to use the validated SGA to assess nutritional status of patients undergoing surgical management for HNC, which is notably lacking in similar studies. This, in conjunction with being undertaken by experienced professionals trained in the use of this tool, adds weight to our findings on the role of malnutrition in a HNC surgical population. Limitations of this study include being a retrospective study design, a single site study, a small sample size and a patient cohort > 10 years old. This may result in selection bias and limit generalizability; however, demographics of this cohort are similar to others reported in the literature and the surgical procedures are still part of contemporary management so these potential effects may be minimized. The small sample size limited the multivariate analysis that was able to be performed to enable adjusting for any confounding variables and also limited the power of the statistical analysis to be able to detect a significant difference.

In conclusion, this study found that having a worse nutritional status pre-operatively increased the risk of experiencing post-operative complications and increased length of hospitalization. Greater emphasis for the optimization of pre-operative nutritional status should be given to patients with HNC planned for major surgery, given the effects malnutrition has on the post-operative course. Therefore, routine nutrition screening and early referral for individualized nutrition support is recommended.

## Data Availability

The data that supports the findings of this study are not openly available due to the HREC approval not supporting this, however are available from the corresponding author upon reasonable request.
